# Genome and Epigenome Disorders and Male Infertility: Feedback from 15 Years of Clinical and Research Experience

**DOI:** 10.3390/genes15030377

**Published:** 2024-03-19

**Authors:** Debbie Montjean, Marion Beaumont, Abdelhafid Natiq, Noureddine Louanjli, Andre Hazout, Pierre Miron, Thomas Liehr, Rosalie Cabry, Ilham Ratbi, Moncef Benkhalifa

**Affiliations:** 1 Fertilys Fertility Centers Laval and Brossard, 1950 Maurice-Gauvin Street, Laval, QC H7S 1Z5, Canada; debbie.montjean@fertilys.org (D.M.);; 2 Genetics Department, Eylau/Unilabs Laboratory, 92110 Clichy, France; marion.beaumont@unilabs.com; 3 Center for Genomics of Human Pathologies (GENOPATH), Faculty of Medicine and Pharmacy, University Mohammed V of Rabat, Rabat, Moroccoi.ratbi@um5r.ac.ma (I.R.); 4 National Laboratory Mohammed VI, Mohammed VI Foundation of Casablanca, Casablanca, Morocco; 5 Andro-Genetics Unit, Labomac, Casablanca, Moroccohazout.andre@hotmail.fr (A.H.); 6 Institute für Humangenetik, Universitätsklinikum Jena, Friedrich Schiller Universität, 07743 Jena, Germany; 7 Reproductive Medicine, Reproductive Biology & Genetics, CECOS Picardie, University Hospital & School of Medicine, Picardie University Jules Verne, 80000 Amiens, France; 8 PeriTox Laboratory, Perinatality & Toxic Risks, UMR-I 01 INERIS, Picardie University Jules Verne, 80000 Amiens, France; 9 Medical Genetics Unit, Ibn Sina University Hospital Center, Rabat, Morocco

**Keywords:** male infertility, genome and epigenome disorders

## Abstract

Infertility affects around 20% of couples of reproductive age; however, in some societies, as many as one-third of couples are unable to conceive. Different factors contribute to the decline of male fertility, such us environmental and professional exposure to endocrine disruptors, oxidative stress, and life habits with the risk of de novo epigenetics dysregulation. Since the fantastic development of new “omes and omics” technologies, the contribution of inherited or de novo genomes and epigenome disorders to male infertility have been further elucidated. Many other techniques have become available to andrology laboratories for the investigation of genome and epigenome integrity and the maturation and the competency of spermatozoa. All these new methods of assessment are highlighting the importance of genetics and epigenetics investigation for assisted reproduction pathology and for supporting professionals in counselling patients and proposing different management strategies for male infertility. This aims to improve clinical outcomes while minimizing the risk of genetics or health problems at birth.

## 1. Introduction

Fertility decline is a worldwide problem, affecting an average of 19–20% of couples and up to 30% in some countries. Male factor may contribute to up to 40% of infertility cases. There are several causes of male infertility, such as endocrine disorder, urogenital abnormalities, immunological disorders, sexual dysfunction, and primary testicular defects with altered spermatogenesis. However, up to 20% of cases remain unexplained (idiopathic). Male fertility assessment is commonly performed by standard semen analysis according to the World Health Organization (WHO, 2021) [1]. However, the limits of this analysis are being questioned and complementary testing is frequently suggested to refine male fertility investigation. Indeed, although the spermiogram remains the gold standard test for male fertility evaluation, it does not assess sperm competency, nor does it identify other underlying causes of male infertility such as genetic and epigenetic disorders.

In reproductive pathology investigation practice, nearly 20% of cases of genetic counselling are related to fertility problems with at least 15% involving men with issues from basic karyotype abnormalities to specific or multiple gene disorders with adverse effects at proteomic levels.

Based on his observation, one can admit that there is a growing need for the inclusion of new sperm examination methods for evaluating genome and epigenome integrity as well as the type and extent of eventual key proteins. In parallel to the somatic genome investigation of infertile men, there is evidence that coupling conventional semen analysis with spermatozoa genomic and epigenetic assays would improve the current state of patient counselling and, eventually, treatment before an assisted reproductive technology cycle.

**Figure 1:**
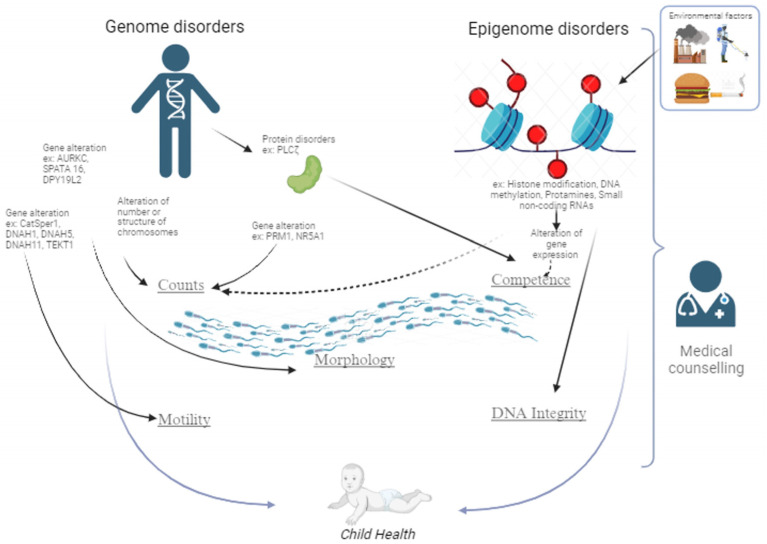
Genetic and epigenetic disorders impacting male fertility. Hard lines reflect proven impact. Dotted lines reflect association.

This article aims to summarize advances made over the past decades in knowledge about genetics and epigenetics factors in the context of male infertility investigation. Examples of genetic and epigenetic factors are available in Figure 1, which depicts the impact of genetics and epigenetic disorders on commonly assessed sperm parameters, namely, counts, motility, and morphology. Also included are crucial sperm parameters that are not assessed by routine semen analysis, namely, sperm competence, which reflects the sperm’s ability to fertilize and give rise to a viable embryo, and sperm DNA integrity, which is usually evaluated by complementary assays (chromatin dispersion and DNA fragmentation assays). The figure also includes the link between genome and epigenome disorders and the health of born children, which highlights the importance of medical counselling, particularly when lifestyle factors may be improved.

## 2. Genetics and Male Infertility

Genetic causes of male infertility are found in up to 10% of cases, mainly in cases of severe quantitative infertility defects, whereas 40–60% of cases with spermatogenic impairment remain unexplained and, among moderate oligozoospermia cases, this fraction is close to 80% [2]. The extreme clinical and genetic heterogeneity of male infertility and the reduced reproductive fitness of affected males are two major challenges for the identification of new causative genetic factors.

The karyotype remains the gold standard for genetic evaluation in the field of reproductive biology. Although of low resolution, it identifies numerical or structural chromosome abnormalities in nearly 12% of infertile couples, with about 6% of all male infertility-associated anomalies, such as reciprocal and Robertsonian translocations. Klinefelter’s syndrome (XXY), a numerical chromosome defect, is common and accounts for nearly 14% of all non-obstructive azoospermia. Numerical and structural chromosomal changes are responsible for meiotic errors, ultimately resulting in spermatogenesis alteration.

Additional molecular analyses are required, even routinely, to identify genetic variants in both the Y chromosome and the autosomes of infertile men.

The Y chromosome is largely investigated in the context of male infertility. Indeed, it carries multiple genes that have been demonstrated to be essential for the differentiation of the male gonad and for male germ cell development. For example, the *SRY* gene, which is located on the short arm of the Y chromosome, encodes for a crucial protein that plays a main role in the regulation of testis formation during embryogenesis. Germ cell migration, differentiation, and function are also under the control of genes located on the Y chromosome. The existence of a factor controlling spermatogenesis in the distal portion of the long arm of the Y chromosome (Yq11) was suggested in the late 1970s, since a large deletion of this portion was observed in six azoospermic men [3]. Nowadays, Y chromosome microdeletions are well characterized. Y chromosome mapping includes a subdivision of the long arm into three non-overlapping regions called the AZF (AZoospermic Factor): AZFa, AZFb, and AZFc. These regions contain the genes required for spermatogenesis. Complete AZFa deletion (1% of the AZF deletions) is associated with the most severe phenotype: Sertoli Cell Only Syndrome (Azoospermia). Nevertheless, deletions within the AZFa region are described in cases with variable phenotypes ranging from azoospermia to normospermia. As an example, a partial deletion of AZFa resulting in the absence of the USP9Y gene was reported in a normospermic man [4]. This observation suggests that USP9Y, once considered a candidate gene for infertility and azoospermia, is not essential for male reproduction and that the AZFa phenotype is likely to be a consequence of the absence of DBY (DEAD-box RNA helicase Y) [5].

Based on these observations, the screening for deletions in these regions is highly suggested by practitioners in cases with diminished sperm counts. Indeed, it allows for the detection of 95% of the interstitial submicroscopic deletions in azoospermia and oligozoospermic men (<5 million spermatozoa/mL).

To bridge the gap between AZF microdeletions and karyotypes, Kalantati and colleagues retrospectively analyzed 10,388 patients with disruptive spermatogenesis, either non-obstructive azoospermia (NOA) or severe oligospermia. They concluded that all chromosomally abnormal NOA cases, except males with a 46, XY/45, or X karyotype, were not indicated for AZF screening. On the other hand, the case with Inv(Y) (p11.2q12); isodicentric idic(Y) (q11.2) or idic(y) (p12.2); ring chromosome r(Y); and derivatives as der (y) should also be referred for AZF deletion screening. The authors showed that only 1% of cases with a sperm count > 1 × 10^6^/mL had Y-chromosomal microdeletions [6]. There is a need to identify relevant sperm count thresholds as an indicator for Y chromosome investigation, to maintain the cost-effectiveness of such investigations [6]

Although the conventional karyotype remains a key source of information, other technologies with a higher resolution will surely improve diagnostics and counselling in the future.

Studies in animal models have identified several hundred candidate genes involved in spermatogenesis [7]. Nevertheless, only a limited number of these genes were found to be mutated in infertile men. Advances in next generation sequencing technologies have been a significant asset in the identification of novel genes responsible for a large panel of human pathologies. Similarly, the application of next generation sequencing to male infertility allowed for the identification of several new genetic factors [2]. The last published standardized clinical validity assessment of monogenic causes of male infertility reported 120 genes that are linked to 104 infertility phenotypes [8]. This valuable report is a starting point for an update of standardized international guidelines for clinical genetic testing in male infertility in the era of new genome sequencing by developing well-standardized targeted gene panels.

The identification of the specific genetic cause of male infertility is relevant to the optimization of the clinical treatment and management of correctable conditions in infertile men and to help them avoid unnecessary interventions. It is also helpful for the selection of the best assisted reproductive technology [8]. It also helps in providing appropriate genetic counseling about the risk of the transmission of infertility to the next generation and potential comorbidities [9].

## 3. Sperm Genome Decays

### 3.1. DNA Fragmentation

A low physiological level of reactive oxygen species (ROS) is required for normal sperm function, but if ROS levels exceed standards, they lead to the deterioration of the function of spermatozoa [10]. Unlike somatic cells, spermatozoa are very vulnerable to ROS because their membrane structure has a limited amount of oxidative stress-protective enzymes. Sperm DNA breaks (single-stranded or double-stranded) occur essentially due to oxidative stress (post-testicular), but may also be caused by the apoptotic intra testicular activity that can be provoked by hyperthermia (varicocele), infection (chronic prostatitis), age, or the chronic use of toxic substances (e.g., tobacco, cannabis) [10,11].

It has been proven that sperm with high DNA fragmentation are able to fertilize oocytes with the same efficiency as sperm without fragmentation. Furthermore, even an apparently normal sperm may have nuclear DNA damage [12,13]. However, if sperm nuclear DNA is damaged, it can lead to errors in DNA replication, transcription, and translation during embryogenesis, after its incorporation into the embryonic genome [14]. Nowadays, there are sufficient data confirming a negative effect of the use of sperm cells with fragmented DNA. Therefore, elevated DNA fragmentation may be considered pathological [15].

This has been supported over the past years by the increasing number of studies evaluating the input of sperm DNA fragmentation analysis during male fertility work-up and the clinical utility of this analysis. Indeed, elevated sperm DNA fragmentation was shown to impact spontaneous fertility with a longer time to achieve pregnancy and increased risk of pregnancy loss [16,17]. In addition, assisted reproductive technology outcomes were also reported to be influenced by sperm DNA fragmentation level. In fact, elevated sperm DNA fragmentation level was associated with lower chances of success after IUI, lower fertilization rate, lower embryo cleavage rate, lower implantation rate, and, in turn, decreased live birth rate [10,18,19,20,21,22,23]. Based on this evidence, one can admit that sperm DNA fragmentation assessment has significant value for male fertility evaluation.

### 3.2. Sperm Chromatin Decondensation

During spermatogenesis, more than 80% of the histones are replaced by protamines, leading to a tight compaction of the sperm chromatin. Two types of protamines have been studied in the context of male infertility, namely, Protamine 1 and Protamine 2. A ratio close to 1 reflects a good quality of chromatin compaction [24]. Any disjunction of the chromatin condensation can potentially result in defects in fertilization and early embryonic development [24]. These may materialize in IVF/ICSI embryonic cell stage blocking before or after genome activation or result in spontaneous miscarriages. The mechanism of the origin of sperm DNA decondensation is still poorly known. The failure of sperm chromatin condensation or premature chromatin decondensation exposes sperm DNA to an increased risk of DNA fragmentation [24].

Truthfully, the paternal genome in sperm is condensed in a specific way, certainly to protect DNA during the transit of the sperm to the oocyte, before fertilization. The existence of this unique packaging of chromatin has a significant impact on fertility and embryonic development. A sperm DNA decondensation rate higher than 30% is considered abnormal by some researchers while others consider the cut-off to be 20% [12,13]. No consensus has been found in terms of a cut-off value, and standardized protocols are still required. However, sperm chromatin dispersion provides informative insight to aid the prediction of assisted reproduction outcomes.

### 3.3. Sperm Parameters Declining and Specific Genes Defects

#### 3.3.1. Reduced Sperm Counts

Many genes have been investigated in the context of male infertility; monogenic variants are correlated with disruptive spermatogenesis, resulting in reduced sperm counts and male infertility, for instance PRM1, NR5A1, MTHFR, and MTSR. Sperm protamine 1 (PRM1) is one of the basic proteins that replaces histones during spermiogenesis. The impact of mutations or variants in the protamine genes on male fertility has raised considerable interest [25]. Variants in PRM1 were documented and were found to be associated with oligozoospermia [26]. Another example is a member of the nuclear subfamily, group A: NR5A1. This gene encodes for a 461 amino acid protein belonging to the nuclear receptor superfamily. NR5A1 is a key transcriptional regulator of genes involved in the hypothalamic–pituitary–steroidogenic axis. It is expressed in developing gonads and its expression is maintained during adulthood. Mutations in NR5A1 cause a large spectrum of phenotypes including partial and complete gonadal dysgenesis, penoscrotal hypospadias, and micropenis [27]. NR5A1 mutations have also been described in 4% of men with severe spermatogenic defects [28].

Methylentetrahydrofolate reductase (MTHFR) encodes for a key enzyme that is involved in the folate metabolism pathway. MTHFR also plays a major a role in DNA, protein, and phospholipid methylation [29]. Lastly, MTRR encodes for the protein MTSR, activates methionine synthase, and has also been shown to play a role in spermatogenesis disruption. Human spermatogenesis can be affected by changes in folate status via DNA methylation and, in turn, gene expression. One other way that folate affects spermatogenesis is by inducing errors during DNA synthesis, leading to errors in DNA repair, strand breakage, and possibly chromosomal anomalies. Although the involvement of MTHFR, MTRR, and Hcy in spermatogenesis decay has been proven, the relationship between the variants in these genes and spermatogenesis is still not conclusive [30,31].

#### 3.3.2. Asthenozoospermia

Isolated asthenozoospermia is a rare feature that is frequently associated with genetic errors. Few genes have been studied in this context. As an example, *CatSper1* and *CatSper2* (cation channel of sperm 1 and 2) regulate intracellular calcium channels and potassium currents in sperm. The alteration of *CatSper* genes expression level and proteins was reported to negatively impact sperm motility [32].

Other genes are associated with asthenozoospermia, for instance, *DNAH1*, *DNAH5*, and *DNAH11* (dynein, axonemal, heavy chain 1, 5, 11), which encode axonemal proteins, and *TEKT1*, which is an α helical protein required for flagella assembly [33].

#### 3.3.3. Teratozoospermia

Aurora Kinase C (*AURKC*), which exhibits a high expression level in the testis, is involved in cytokinesis, mitosis, and meiosis. The deletion of a cytosine in exon3 (c.144delC) is associated with large-headed, multiflagellar polyploidy spermatozoa. The prevalence of this mutation is high in the North African population [34]. Spermatogenesis-associated 16 (*SPATA16*) is specific to the human testis and may play a role in acrosome formation during spermiogenesis. The mutation in exon4, c.848G>A, is predicted to result in the p.R283Q amino acid change located at the C-terminal end, and it is associated with globozoospermia (blockage of sperm head elongation and acrosome formation) [35]. A last example of monogenic disorder inducing teratozoospermia is *DPY19L2*; it encodes for an uncharacterized protein, the deletion of which causes male infertility due to globozoospermia [36].

#### 3.3.4. Protein Dysregulation

Sperm proteins have been investigated in the past decade, and protamines (see Section 3.3.5.2) and PLCz turned out to be of particular interest. In the context of the low fertilization potential of sperm, the phospholipase C Zeta protein (PLCζ) is gaining interest for clinical applications. PLCζ is a protein located in the equatorial and acrosomal regions of spermatozoa. It is a crucial player in the initiation of the signaling cascade, marking the first steps of fertilization. Indeed, it induces the intracellular calcium (Ca^2+^) oscillations necessary for the activation of oocytes after fertilization [37,38,39]. During fertilization, PLCζ is released into the cytoplasm of the oocyte and induces Ca^2+^ oscillations via the inositol 1,4,5-triphosphate signaling pathway [38].

In cases of the absence (KO) or deficiency of sperm in PLCζ, in animal models, although sperm parameters looked unchanged, the rates of fertilization and early embryonic development were negatively impacted, with an inability to induce Ca^2+^ oscillations [40]. In addition, the microinjection of PLCζ protein or mRNA into mouse eggs corrected the phenotype observed in KO mice, restoring normal fertilization and embryonic development [41,42,43].

The evaluation of PLCζ protein levels in spermatozoa during a male infertility work-up could then help to detect patients at risk of poor fertilization or total fertilization failure. The affected patients may not display any alteration in their sperm parameters. Therefore, PLCζ protein levels will provide information about sperm competence and will help with patient counselling and management. Indeed, men in whom low levels of PLCZ are detected would be advised to proceed with in vitro fertilization rather than IUI. In such cases, artificial oocyte activation could be induced artificially (mechanically or chemically). This method is expected to significantly improve fertilization and oocyte activation rates in patients with PLCζ deficiency [44,45,46]. The identification of a pathological threshold value of PLCζ is in progress.

#### 3.3.5. Epigenetic Marks

The term “epigenetics” encompasses the modifications that regulate gene expression without changing the underlying DNA sequence. Epigenetic changes may be considered the link between environmental factors and genetics. Epigenetic marks in mature spermatozoa include post-translational histone modifications (PTM), protamines, small non-coding RNA, and DNA methylation, and possibly the architecture of sperm nuclei. Epigenetic modifications can be considered as a network that aims to establish and maintain genes’ expression status [47].

##### 3.3.5.1. Histones

Histones are alkaline proteins involved in packaging DNA into structural units called nucleosomes. Nucleosomes are octameric complexes composed of two copies of each of the core DNA-binding histones, H2A, H2B, H3, and H4. Histones H3 and H4 have long tails protruding from the nucleosome and are subjected to PTM. PTM includes ubiquitination, lysine acetylation, lysine and arginine methylation, and serine and threonine phosphorylation, and can change the interaction of histones with DNA. Gene expression is governed by the methylation, acetylation, ubiquitination, and phosphorylation of the histone, depending on the position and type of the modification of the amino acid involved [48]. During spermiogenesis, spermatids replace 90–95% of their histones with protamines. Residual histones remain subjected to PTM, which seems to be crucial for spermatogenesis and early embryonic development [49]. Indeed, they have a direct impact on DNA packaging and thus on the competence of sperm to give rise to a viable embryo [49]. In fact, changes in PTM are associated with sperm defects. For instance, the distribution of site-specific modifications of histones like H3K9ac, H4ac, or H3K4me was shown to be significantly different in infertile men with impaired sperm count, motility, and chromatin maturity as compared to normospermic fertile men with a putative subsequent impact on pregnancy outcome [50,51,52,53]. In addition, animal models shed light on the importance of H3K4me in sperm by showing a dramatic impact on offspring development and survival in a transgenerational manner, when H3K4me is disrupted in the developing sperm of the father [54]. The association between PTM and male infertility has mainly been observed in animal models, and research involving humans is still ongoing.

##### 3.3.5.2. Protamines

During spermatogenesis, sperm cells undergo protamination, which is the replacement of 90–95% of the core histones surrounding the DNA with small basic arginine-rich nuclear proteins, called protamines (protamine 1: P1 and protamine 2: P2). This process leads to a highly condensed and transcriptionally silent sperm chromatin. This high level of chromatin condensation is crucial for sperm motility and DNA integrity, since it protects the sperm genome against endogenous and exogenous agents such as nuclease, free radicals, and mutagens [55]. Any modification in the protamine structure or ratio may be associated with higher sperm DNA fragmentation, decreased fertilization rate, and altered sperm motility and/or morphology, ultimately resulting in subfertility or even infertility [49]. Indeed, mutation screening in infertile men revealed point mutations in protamine genes associated with protein changes. These errors negatively affected spermatogenesis and induced elevated sperm DNA fragmentation [56]. Furthermore, an impairment in the histone-to-protamine exchange in the sperm of infertile men with disruptive spermatogenesis was associated with poor in vitro fertilizing ability and faulty subsequent embryo development, likely due to abnormal sperm chromatin compaction [57].

##### 3.3.5.3. RNA Associated Gene Silencing

Small non-coding RNAs bind to complementary mRNA and subsequently induce their degradation to regulate gene expression. A number of small noncoding RNAs have been identified in male germ cells, and a disruption of their pathway may lead to spermatogenic failure [58,59]. However, the association between non-coding RNAs and male infertility is still under investigation and the amount of data on this aspect is increasing. A possible correlation between the expression profile of non-coding RNA and sperm quality was mentioned in a pilot study. The authors suggested that non-coding RNA could be used as a potential biomarker [60]. Recently, small non-coding RNAs were assessed clinically in the context of male infertility. Some micro-RNAs were suggested to be potential biomarkers for infertility diagnosis as well as for other diseases like varicocele, and predictors of surgical and in vitro fertilization outcomes [61].

#### 3.3.6. Methylome Unbalance

The establishment of DNA methylation in the male germline is not only important for ensuring normal sperm function but also because it contributes to embryonic development and, in turn, impacts the health of the children born. A decreased level of methylation (also known as hypomethylation) of sperm DNA is associated with altered testicular histology, reduced sperm production, and male infertility [62,63]. The preliminary observations made in rodents were also described in humans. Indeed, the methylation of sperm DNA was also found to be altered in the sperm of men exposed to environmental factors as well as in men suffering from infertility [64,65,66,67,68,69,70,71,72,73]. Further investigations showed that these alterations in the methylation profile can affect the entire genome [65,72].

However, the trend of global changes in sperm DNA methylation in infertile men compared to normospermic controls is still open to debate. The contradictory nature of the results obtained (hyper- vs. hypomethylation) calls for new investigations to improve the characterization of the changes in DNA methylation associated with disrupted spermatogenesis and to explain the biological mechanisms and the clinical impacts of these epigenetic errors.

Analysis of the sperm methylation profile will certainly have its place in the diagnosis of male infertility because an alteration can lead to potential sperm defects associated with fertility disorders. In addition, an alteration in the methylation profile can be transmitted transgenerationally to the offspring, leading to the heredity of various pathologies such as spermatogenesis defects, male infertility, and other non-reproductive pathologies like breast cancer and kidney, prostate, and immune dysfunctions [74,75].

#### 3.3.7. Nuclear Architecture Disorganization

The three-dimensional organization of nuclear DNA has been shown to play a role in the gene expression of different cell types [76]. Still, mechanistic insights into how this works in detail are far from being understood. Even though the nuclear architecture of normal human sperm is quite similar to other genetically active cells like fibroblasts or lymphocytes [77], studies on the effects of supernumerary normal or marker chromosomes have still not been systematically performed [78]. This is unfortunate, as, e.g., the presence of small supernumerary marker chromosomes (irrespective of its chromosomal origin and genetic content) may lead to male infertility, specifically to oligoastenoteratozoospermia [79].

#### 3.3.8. Peripheral Free Circulating DNA and Sperm Parameters

Data on cf-DNA levels in the seminal plasma of men with sperm alterations have not previously been well documented or published. The presence of free nucleic acids in seminal plasma has also not been well documented. Li and colleagues showed that seminal cf-DNA levels in azoospermic patients were significantly higher than in individuals displaying normal sperm parameters [80]. Costa and collaborators suggested that elevated seminal cf-DNA levels are related to defects in sperm motility and morphology. In 2018, an association between seminal plasma cell-free mitochondrial copy number and sperm parameters was observed. A comparison between men with abnormal sperm characteristics and normospermic controls showed higher mean cf-DNA levels in patients with sperm abnormalities than in controls. The cf-DNA levels were shown to be significantly higher in men with azoospermia and men with teratozoospermia. Although the study group numbers were small, these results may open up new diagnostic and prognostic perspectives for male infertility [81].

## 4. What for the Future

The evidence of the potential for epigenetic sperm alterations to affect embryogenesis and IVF outcome is clear. Indeed, when comparing IVF/pre-implantation genetic screening couples with altered sperm parameters to female age-matched controls, elevated rates of miscarriage and altered embryo DNA methylation and gene expression were described. These results suggest an association between altered embryo methylomes and gene expression abnormalities, highlighting the important role of sperm epigenetics [82]. Inherited or de novo, epigenetic disorders can also produce problems in embryo development, implantation failures, miscarriages, and risks of imprinting diseases at post-natal stages (pre-pubertal and adult stages) as well. To date, the genetic and epigenetic factors of male infertility have been well documented, but further studies will help to clarify the implications for, and the role of, epigenetic marks in the onset of spermatogenic disruption. It is of particular interest to focus additional studies on the effects of environment and ageing on the epigenetic landscape in sperm and how they lead to male infertility.

It is time to revisit our practice and services to support clinicians and patients for better clinical management and therapy. In the daily practice of male infertility investigation, it is important to improve our approach to sperm investigation by adding genome and epigenome dysfunction analyses because paternal genetic and epigenetic disorders are contributing to the current decline of male fertility. Technological developments such as next genome sequencing, optical genome mapping, and multi-omics will help to provide new data and improve patient counselling [83]. However, the financial and technical limits of performing such a large range of tests in routine practice need to be considered. Finally, as depicted in Figure 1, it is important to keep in mind the important impact of environmental factors on male fertility and to advise patients to correct them when possible.

## Data Availability

No new data were created.
